# Establishment of two basal-like breast cancer cell lines with extremely low tumorigenicity from Taiwanese premenopausal women

**DOI:** 10.1007/s13577-017-0197-3

**Published:** 2018-02-26

**Authors:** Wen-Ling Kuo, Shir-Hwa Ueng, Chun-Hsing Wu, Li-Yu Lee, Yun-Shien Lee, Ming-Chin Yu, Shin-Cheh Chen, Chi-Chang Yu, Chi-Neu Tsai

**Affiliations:** 1 Division of Breast Surgery and General Surgery, Department of Surgery, Chang Gung Memorial Hospital, Linkou, Guishan Dist., Taoyuan, 33305 Taiwan; 2 Graduate Institute of Clinical Medical Sciences, Chang Gung University, Taoyuan, 33302 Taiwan; 3 Department of Pathology, Chang Gung Memorial Hospital, Chang Gung University, Guishan Dist., Taoyuan, 33305 Taiwan; 4 Department of Biotechnology, Ming Chuan University, Guishan Dist., Taoyuan, 33348 Taiwan; 5 Genomic Medicine Research Core Laboratory, Chang Gung Memorial Hospital, Linkou, Guishan Dist., Taoyuan, 33305 Taiwan; 6 Department of Pediatric, Chang-Gung Memorial Hospital, LinKou, Guishan Dist., Taoyuan, 33305 Taiwan

**Keywords:** Premenopausal breast cancer cell lines, Basal-like breast cancer, Low tumorigenicity

## Abstract

The research of carcinogenetic mechanisms of breast cancer in different ethnic backgrounds is an interesting field, as clinical features of breast cancers vary among races. High premenopausal incidence is distinctive in East-Asian breast cancer. However, human cell lines derived from Asian primary breast tumor are rare. To provide alternative cell line models with a relevant genetic background, we aimed to establish breast cancer cell lines from Taiwanese patients of Han-Chinese ethnicity. Fresh tissue from mammary tumors were digested into organoids, plated and grown in basal serum-free medium of human mammary epithelial cells (HuMEC) with supplements. Cells were further enriched by positive selection with CD326 (epithelial cell adhesion molecule; EpCAM)-coated micro-magnetic beads. Two breast cancer cell lines derived from premenopausal women were successfully established by this method, and named Chang-Gung Breast Cancer 01 (CGBC 01) and 02 (CGBC 02). These two cell lines had a similar phenotype with weak expression of estrogen receptor (ER), progesterone receptor (PR), and without amplification of receptor tyrosine protein kinase erbB-2 (HER2/neu). Genome-wide Single Nucleotide Polymorphism (SNP) array showed multiple copy number alterations in both cell lines. Based on gene expression profiles, CGBC 01 and 02 were clustered into basal-like subtype with reference to the breast cancer cell line gene expression database. The tumorigenicity of both cell lines was extremely low in both anchorage-independence assay and transplantation into the mammary fat pads of nude mice. CGBC 01 and CGBC 02 are low tumorigenic breast cancer cell lines, established from Han-Chinese premenopausal breast cancer patients, which serve as in vitro models in studying the biological features of Asian breast cancer.

**Electronic supplementary material:**

The online version of this article (10.1007/s13577-017-0197-3) contains supplementary material, which is available to authorized users.

## Introduction

Breast cancer is the most common malignancy affecting women globally. It is the second common cause of cancer-related death in women and is one of the most extensively studied cancer types. Among the in vitro breast cancer research models, cell lines are the most widely used tools in laboratory studies of mechanisms of cancer growth, metastasis, tumor microenvironment crosstalk, signaling pathways, drug screening, and functional genomics [1, 2]. Currently, there are approximately 70 breast cancer cell lines established from either primary tumors or malignant pleural effusions (Supplementary Table 1). Most of these cell lines could be categorized into at least five molecular subtypes based on gene expression signatures: luminal A, luminal B, basal-like, HER2-enriched, and claudin-low [1–4]. Since breast cancer is a heterogeneous disease comprising different subtypes with different characteristics, the breast cancer cell line used should be specific to the subtype being studied [1–5].

Luminal A (ER+, PR+/−, HER2−) and B (ER+, PR+/−, HER2+) breast cancers are characterized by estrogen receptor (ER) expression with or without progesterone receptor (PR) [6–8]. Examples of widely used luminal A cell lines are MCF7 and T-47D. Luminal B subtype cancer cells, such as BT-474 and ZR-75, are also ER positive, but display high proliferation index [4]. HER2 (ErbB-2 receptor tyrosine kinase 2) overexpression is a signature of HER2-enriched breast cancer (ER−, PR−, HER2+) cell lines. SK-BR-3, MDA-MB-361 and MDA-MB-453 are examples of HER2 type cell lines [1, 4, 7, 9]. Basal-like breast cancers are characterized by ER−, PR−, HER2−, cytokeratin 5/6 (CK5/6)+ and expression of epidermal growth factor receptor (EGFR). MDA-MB-468 is an example of basal-like cell lines [3, 4, 7]. Claudin-low subtype (ER−, PR−, HER2−, claudin-low, E-cadherin low) represents the clinical triple-negative breast cancers showing enrichment of epithelial-to-mesenchymal transition markers and cancer stem cell-like features with unfavorable outcomes [10]. One example of claudin-low cell line is MDA-MB-231 [4, 11, 12]. Each subtype of breast cancer can be viewed as a distinct type of disease rather than a variant of the same cancer with minor differences.

Most of the cell lines used commonly in breast cancer research, including the abovementioned, were derived from Western countries. Most of continuous breast cell lines derived from tumors at metastatic sites such as pleural effusion represent cancers at more advanced stages of tumor development [1, 3, 4, 6–9, 11] (Supplementary Table 1). Even though there are reports of differences in breast cancer based on the epidemiological, clinical and genetic factors [13–17], it is difficult to assess the effect of ethnic background on the cancer phenotype. The phenotype of breast cancer in Asians has rarely been characterized or discussed as much as that in African-American or Caucasian women [18]. The incidence of breast cancer in Asia is significantly lower than that in Western countries. However, there is an emerging trend of increasing incidence in premenopausal Asian female under the age of 50 [19–22]. In contrast, the incidence of breast cancer only increases with age after menopause in Caucasian women [23, 24]. The factors contributing to such differences in the age of onset in Asian women may include genetics, Westernized life style, obesity, and exposure to xenoestrogen from the environment [13, 14, 16, 19, 20, 25, 26]. Features in prognosis, cancer cell metabolism and immune cell populations in Asian young breast cancer were being more characterized recently [27–30]. These clinical observation and hypothesis need to be testified in relevant models. However, few breast cancer cell lines derived from Asian people are publicly available (Supplementary Table 1). In order to gain insights into the etiology and development of Asian breast cancer, we aimed to establish breast cancer cell lines derived from Taiwanese women to provide alternative cell line models with a relevant genetic background. Two continuous cell lines, CGBC 01 and CGBC 02, have been successfully established in this study from the primary mammary carcinoma of two premenopausal women. The cell lines have been subcultured more than 70 passages (P70) in our laboratory. The characteristics, classification, molecular profiling and tumorigenicity of these two cell lines are described in this paper.

## Materials and methods

### Patients and tumor collection

The study was approved by the Institutional Review Board (IRB) of Chang Gung Medical Foundation (IRB number: 96-0887B, 97-1592A3, and 97-1639D). Informed consents were obtained from all patients prior to study participation. Total 50 tissue specimens were collected for establishment of primary cell cultures. Fresh breast cancer tissues were collected at surgery from primary tumors in 27 patients with untreated breast cancer (adjacent normal breast tissues were also collected at the same time in 13 cases), and 3 patients with neoadjuvant chemotherapy-treated breast cancer. Normal breast tissues were collected from 3 patients receiving surgery for benign fibroadenoma, and 1 healthy patient receiving reduction mammoplasty. Malignant pleural effusion was collected in another 3 patients with metastatic breast cancers. The demography data of enrolled patient are shown in supplementary Table 2. Patients participating in the study underwent standard preoperative screening according to institutional guidelines, including a detailed medical history, complete physical examination, and whole breast ultrasound. Mammography, chest radiographs, bone scan, and abdominal ultrasound were checked if malignancy was diagnosed.

### Primary tissue culture

Primary cell cultures were set up following procedures described in literature [31, 32]. Fresh tissue pieces from primary mammary tumors were minced with scalpel and scissors followed by chemical dissociation at 37 °C for 3 h in Dulbecco’s modified Eagle medium (DMEM) containing 5% fetal bovine serum (FBS) (Invitrogen, Carlsbad, CA) and 10 mg/mL collagenase A (Roche Applied Science). After digestion, the suspension was centrifuged for 5 min at 10 g and the pellets containing organoids were washed thrice with phosphate-buffered saline (PBS). The organoids were then seeded in six-well CellBIND culture plates (Corning Life Sciences, Lowell, MA) in basal serum-free medium of human mammary epithelial cells (HuMEC) (Invitrogen, Carlsbad, CA) supplemented with bovine pituitary extract (BPE) and cultured at 37 °C in an environment of 5% CO_2_. The final concentrations of the components in the supplemented medium were: 0.4% BPE, 5 μg/mL bovine insulin, 0.5 μg/mL hydrocortisone, and 3 ng/mL recombinant human epidermal growth factor (hEGF). The confluent cells were harvested with 0.05% trypsin–EDTA (Invitrogen, Carlsbad, CA) and subcultured at a split ratio of 1:3, after treatment with trypsin inhibitor (Sigma-Aldrich). 17-β-Estradiol (0.3–1 nM) (Sigma-Aldrich) was added if ER was detected in the primary cancer tissues. The successfully established primary breast cancer cell lines were further enriched with micro-magnetic beads coated with CD326 (also known as epithelial cell adhesion molecule, EpCAM) antibody (MACS, Miltenyi Biotec) [33] before reaching the sixth passage (P6). The cells were adapted to HuMEC and DMEM/F12 (1:1) containing 2 mM l-glutamine (Invitrogen, Carlsbad, CA), 5 μg/mL bovine insulin (Invitrogen, Carlsbad, CA), 3 ng/mL hEGF (Sigma-Aldrich), 0.4 μg/mL dexamethasone (Sigma-Aldrich), and 1 nM 17-β-estradiol, after the 20th passage (P20).

### Cell culture

MCF7 [BCRC No. 60436, Bioresource Collection and Research Center (BCRC), Hsinchu, Taiwan] was maintained in minimum essential Eagle’s medium (MEM) with 2 mM l-glutamine, 0.1 mM non-essential amino acids (Invitrogen, Carlsbad, CA), 1 mM sodium pyruvate (Invitrogen, Carlsbad, CA), and 10% FBS. T-47D (BCRC No. 60250) was maintained in Roswell Park Memorial Institute (RPMI) medium 1640 with 2 mM l-glutamine, 10 mM HEPES (Invitrogen, Carlsbad, CA), 1.0 mM sodium pyruvate, and 10% FBS. MCF 10A (CRL-10317) was purchased from American Tissue Culture Collection (ATCC) and cultured as per the accompanying instructions. The above cell lines were cultured at 37 °C with 5% CO2. MDA-MB-231 (BCRC No. 60425) and MDA-MB-453 (BCRC No. 60429) were purchased from BCRC, which were maintained in Leibovitz’s 15 (L15) medium (Invitrogen, Carlsbad, CA) containing 10% FBS at 37 °C without CO2.

### Flow cytometry

The cells were resuspended at a density of 2 × 10^6^ cells/mL in buffer containing 1× PBS, 2 mM EDTA and 0.5% bovine serum albumin (BSA), and incubated at 4 °C in dark for 10 min with fluorescein isothiocyanate (FITC) conjugated CD326 antibody (MACS, Miltenyi Biotec). After centrifugation and washing, cells were resuspended in 200 μl buffer and analyzed for the expression of EpCAM with FACSCalibur flow cytometer (Becton–Dickinson, Franklin Lakes, NJ), and data were analyzed using ModFit software (Verity Software House, Topsham, ME). At least 20,000 cells were analyzed per sample.

### Immunohistochemical staining (IHC)

For immunohistochemistry, formalin-fixed and paraffin-embedded tissues were sectioned to 4 μm thickness, deparaffinized, rehydrated, and prepared for antigen retrieval. The slides were then incubated at room temperature for 1 h with the following antibodies at appropriate dilution: anti-ER (6F11, Novocastra), anti-PR (1A6, Novocastra), anti-HER2 (polyclone, DAKO), anti-cytokeratin 18 (CK 18) (DC-10, BioGenex), anti-cytokeratin 19 (CK19) (RCK108, BioGenex), anti-CK 5/6 (D5/16 B4, DAKO), anti-EGFR (EGFR.25, Novocastra), anti-E-cadherin (36B5, Novocastra), and anti-EpCAM (polyclonal, Abcam). After incubation, the slides were washed thrice with PBS, incubated with horseradish peroxidase (HRP) conjugated antibody (Zymed) at room temperature for 10 min, and developed by adding 3,3′-diaminobenzidine tetrahydrochloride (DAB) reagent (DAKO) as the chromogen and hematoxylin as the counterstain. Expression of ER and PR was rated using Allred score. All specimens were independently reviewed by two pathologists blinded to the clinical origin of the specimens.

### Western blot

The cells were treated with radioimmunoprecipitation assay (RIPA) lysis buffer, and 25–50 μg of the extracted protein was used for western blotting. The proteins of interest were detected by using the enhanced chemiluminescence system (ECL) (Millipore) as the manufacturer’s instructions. Expression of beta-actin was used as an internal control in this experiment. Antibodies used in this experiment included anti-ER (Abcam, EPR4097), anti-PR (LSBIO, 4E9), anti-EGFR (Cell Signaling), anti-E-cadherin (Millipore), anti-HER2 (Abcam, EP1045Y), anti-CK18 (Abcam), anti-CK19 (Abcam, EP1580Y), anti-CK6 (Abcam, EPR1602Y), anti-CK5 (Abcam), and anti-beta-actin (Sigma-Aldrich).

### Anchorage-independence growth assay

The breast cancer cell lines were seeded in six-well culture plates at a density of 5000 cells/per well in 1.5 mL DMEM containing 10% FBS and 1% UltraPure™ Low Melting Point Agarose (GIBCO) on bottom layer, on top of which was layered 1.5 mL DMEM containing 10% FBS and 0.5% UltraPure™ Low Melting Point Agarose. The final layer consisted of 1 mL of DMEM containing 10% FBS. The cells were incubated at 37 °C for 21–28 days. Colonies were stained with 0.05% crystal violet and colony forming ability of cell lines in soft agar was assessed by the size and the number of colonies.

### RNA extraction, gene expression profiling and processing

RNA was extracted from the breast cancer cell lines using TRizol reagent (Invitrogen, Carlsbad, CA) according to the manufacturer’s instructions, and enriched with RNeasy MinElute kit (Qiagen). The quality and quantity of RNA were analyzed by Agilent Bioanalyzer 2100 (Agilent Technologies, Santa Clara, CA). Ten micrograms of total RNA was reverse transcribed to labeled cDNA using SuperScript II reverse transcriptase (Invitrogen). Twenty-five micrograms of the labeled cDNA was hybridized to probes on GeneChip Human Genome U133A 2.0 Array (http://www.affymetrix.com) in GeneChip Hybridization oven 645 (Affymetrix; Thermo Fisher Scientific Inc.). Following hybridization and washing, gene chip was scanned by GeneChip Scanner 3000 7G4C (Affymetrix; Thermo Fisher Scientific Inc.).

### DNA extraction and genome-wide human single nucleotide polymorphism (SNP) array 6.0

Genomic DNA (gDNA) was extracted from CGBC 01 (P32) and CGBC 02 (P14), respectively, according to the manufacturer’s recommendations (Gentra Puregene blood Kit; Qiagen, Valencia, CA). The extracted genomic DNA was sent to Genomic Medicine Research Core Laboratory of Chang-Gung Memorial Hospital, Linkou, for quality assessment, DNA fragmentation, labeling and hybridization to Affymetrix SNP 6.0 array according to the manufacturer’s protocol (Affymetrix; Thermo Fisher Scientific Inc.). In brief, about 250 ng of gDNA was digested with two restriction enzymes (NspI and StyI). The fragments were ligated to adaptors and amplified by PCR. The PCR products were purified using Agencourt AMPure XP system (Beckman Coulter, Inc.) and quantitated by labeling and hybridization to Affymetrix SNP 6.0 array. The chips were washed, stained, and scanned to generate .cel files for further analysis after overnight hybridization. The .cel files were converted into copy number data file (cnchp) via Genotyping Console™ Software (Affymetrix; Thermo Fisher Scientific Inc.), which was further analyzed to detect chromosome copy number changes via Chromosome Analysis Suite (ChAS; Affymetrix; Thermo Fisher Scientific Inc.).

### Short tandem repeat (STR) DNA profiling

Genomic DNA from cell lines was sent to BCRC of Taiwan for STR profiling and analysis. Amelogenin and 15 STR marker loci were analyzed and compared. The STR profile of each cell line was blasted with the database of other known cell lines, including Hela cells.

### Mice xenograft

Female BALB/c nude mice (BALB/cAnN.Cg-Foxn1nu/CrlNarl) were purchased from the National Laboratory Animal Center (Taipei, Taiwan) and maintained in specific pathogen-free housing and husbandry following institutional reviewed animal protocols. Cancer cell xenografts were injected into the 4th mammary fat pads of mice between the ages of 6 and 8 weeks.

## Results

### Patients and tumor characteristics

Two cell lines, named CGBC 01 and CGBC 02, were successfully established from their primary cell cultures of treatment-naïve breast tumors out of the 33 cell cultures prepared from primary tumors or metastatic cancer cells in pleural effusion (Supplementary Table 2). CGBC 01 (BCRC No. 60610) was deposited in BCRC of Taiwan (http://www.bcrc.firdi.org.tw) and CGBC 02 is under administrative review process for depositing.

**Fig. 1:**
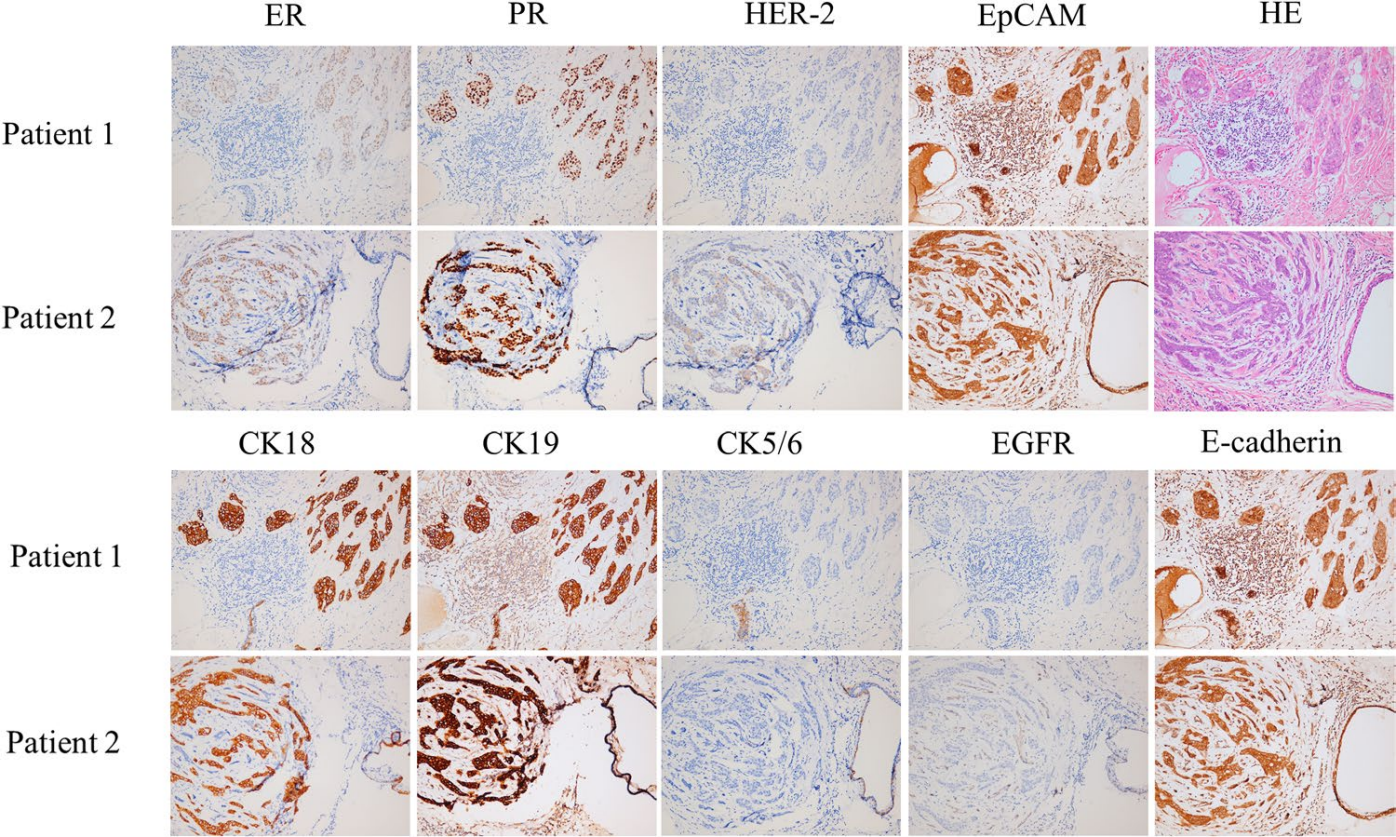
The hematoxylin and eosin, and immunohistochemistry staining of breast cancer tissue sections from patients 1 and 2. The hematoxylin and eosin (H&E) stained tissue section of each patient is shown in the upper right panel; the immunohistochemical staining of ER (estrogen receptor), PR (progesterone receptor), HER2 (human epithelial growth factor receptor 2), EpCAM (epithelial cell adhesion molecule), CK18 (cytokeratin 18), CK19 (cytokeratin 19), CK5/6 (cytokeratin 5/6), and EGFR (epidermal growth factor receptor) in the primary tumor tissue sections are as indicated

CGBC 01 was derived from patient No. 1, a 46-year-old Taiwanese woman with grade II invasive ductal carcinoma. The tumor section was ER (−/+), PR (3 +) and HER2 (−) by IHC scoring (Table 1). CGBC 02 was derived from patient No. 2, a 44-year-old Taiwanese woman with grade III invasive ductal carcinoma and was ER (1 +), PR (3 +) and HER2 (−) (Table 1). Heterogeneous distribution of weakly expressed or absent ER can be observed in the cancer cells across both tumors (Fig. 1). Membrane HER2 expression was very weak and not amplified in both cases (Fig. 1). Epithelial cell adhesion molecule (EpCAM or CD326), also called epithelial-specific antigen (ESA), was strongly present in both tumors. In addition, the luminal cell markers, CK18 and CK19, were also strongly expressed, whereas CK5/6, the basal or myoepithelial cell marker [27], was expressed only in a small number of cancer cells in both cases (Fig. 1). Both cancer patients had no major systemic disease and did not come from hereditary cancer families. Furthermore, the two patients showed on signs of cancer recurrence so far, regularly followed-up 9 years after surgery.

**Table 1:** Clinicopathological characteristics of breast cancer patients enrolled for the establishment of CGBC 01 (patient 01) and CGBC 02 (patient 02) cell lines in this study

Patient no.	Age (years)/menopausal status/family history of breast cancer	Diagnosis/stage	ER	PR	HER2	EpCAM	CK18	CK19	CK5/6	EGFR	E-cadherin
01	46/premenopausal/no	Invasive ductal carcinoma/grade II T2N3M0	**−/+**	**+++**	**−**	**+++**	**+++**	**+++**	**+/−** (focal)	**−**	**+++**
02	44/premenopausal/no	Invasive ductal carcinoma/grade III T2N0M0	**+**	**+++**	**−**	**+++**	**+++**	**+++**	**−**	**+/−** (focal)	**+++**

### Cell propagation

**Fig. 2:**
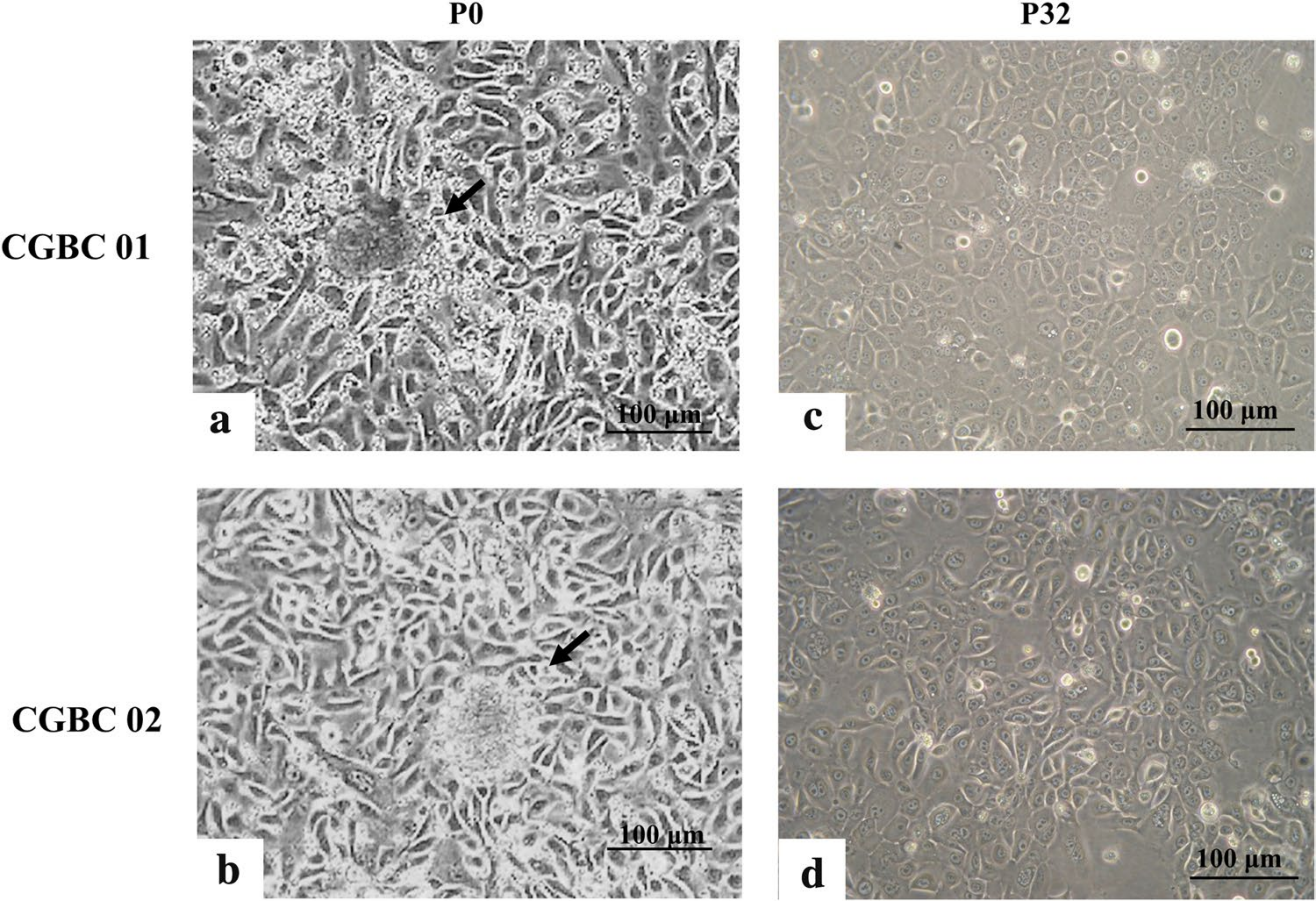
Gross morphology of primary cancer cell cultures and the later established cell lines. **a**, **b** Breast cancer tissues were digested and cultured on tissue culture plates, which formed organoids (P0) 1 week after seeding. Arrows indicate organoids attached to plates with active outgrowth of primary cultured cells. Both primary breast cancer cell lines were further purified by EpCAM microbeads before the sixth passage (P6). **c**, **d** The cancer cells acquired cobble stone to polygonal appearance after multiple passages (P32) (scale bar = 100 µm)

Concentric growth of cells around the organoids that attached to the culture dish (P0) (Fig. 2a) could be observed 1 week after seeding [34, 35]. As shown in Fig. 2, both the size and morphology were heterogeneous in both primary cultures, indicating mixed cellular populations at P0 stage. Cells were subcultured (P1) when they reached confluence 14–21 days after seeding. Outgrowth of elongated, spindle-shaped stromal fibroblasts was suppressed by using serum-free medium, while non-cancerous ductal epithelial cells spontaneously underwent senescence and apoptosis after the first or second subculture, as we had observed in the primary cultures of normal mammary gland (Supplementary Table 2, B_1~4). Cancer cells in CGBC 01 and CGBC 02 survived beyond serial passage in culture and maintained stable proliferation afterwards. Both the cell lines were further enriched by EpCAM selection before the sixth passage (P6). Cellular morphology of both primary cultures became more homogenous after (Fig. 2, right) EpCAM selection than before (Fig. 2, left). Both cell lines have been propagated up to 70 passages in our laboratory and maintained adherent growth, with cuboidal to polygonal shaped cells arranged in cobblestone appearance. The growth curves of both the cell lines, CGBC 01 and CGBC 02, were similar to that of MCF7 and MCF10A; respectively (Supplementary Fig. 1). The cell doubling time of CGBC 01 and 02 was about 30.68 and 52.34 h, respectively, as revealed by cell proliferation assay (Cell Counting kit-8; Dojindo Molecular Technologies, Inc).

### Expression of protein markers in established cell lines

**Fig. 3:**
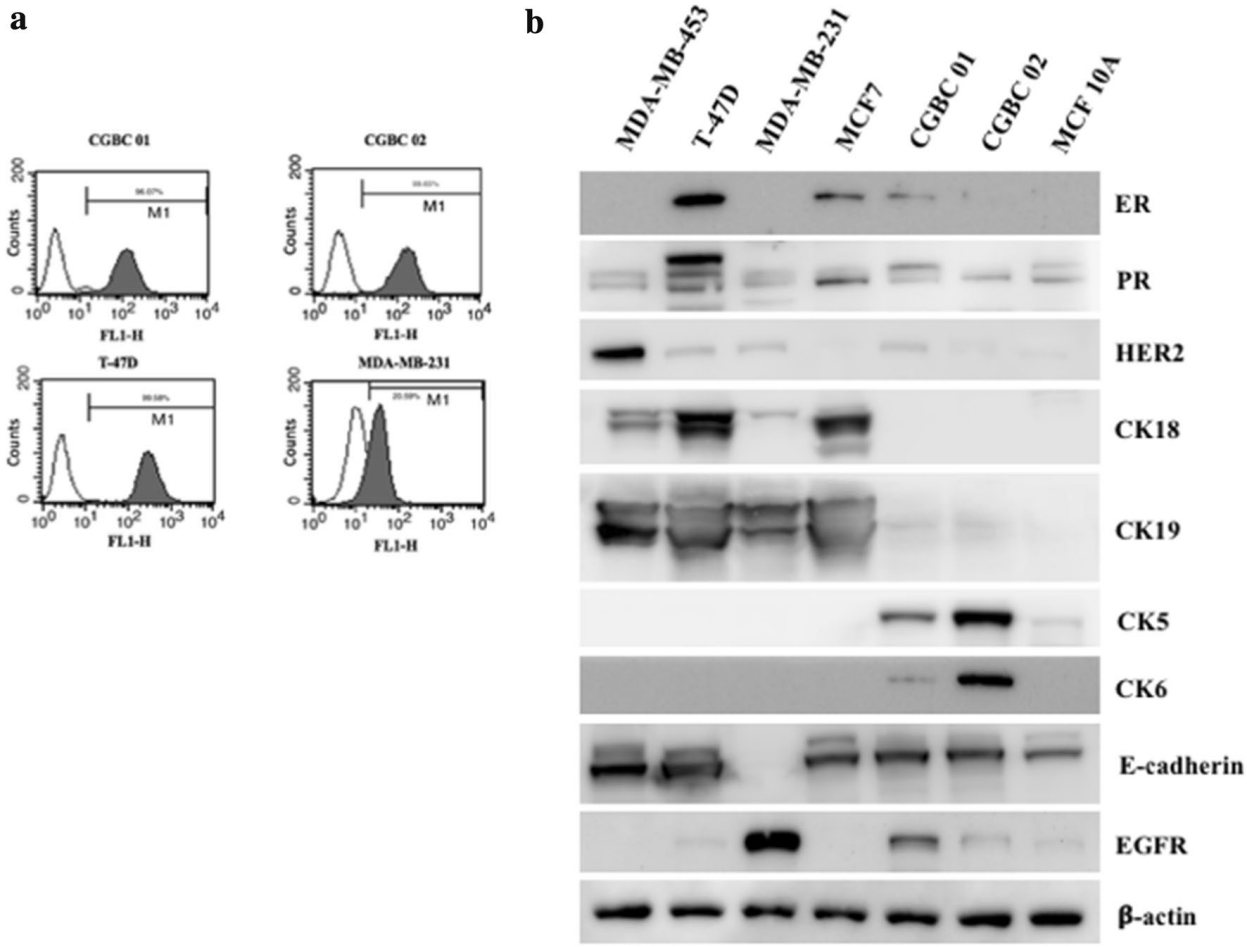
Expression of breast cancer cell markers in CGBC 01 and CGBC 02 cells. **a** Cell surface EpCAM (CD326) was expressed in 96.07, 99.65, 99.58, and 20.59% of CGBC 01, CGBC 02, T-47D, and MDA-MB-231 cells, respectively, as revealed by flow cytometry (**a**). Protein expression of ER, PR, HER2, CK18, CK19, CK5, CK6, E-cadherin, and EGFR in CGBC 01 and CGBC 02 in comparison with other standard cell lines in western blots. The expression of beta-actin was used as internal control in this experiment (**b**)

Since these two cells lines are enriched by positive EpCAM selection, EpCAM was expressed in 96.07% of CGBC 01 and 99.65% of CGBC 02 (Fig. 3a). The expression of ER, PR, HER2, E-cadherin, CK18, CK19, CK5, CK6 and EGFR was analyzed by western blot (Fig. 3b). ER and HER2 were weakly expressed in CGBC 01 and CGBC 02, as is similar to the expression in the original breast tumors from which they derived. The expression of ER in CGBC 01 and 02 was significantly lower than that in the luminal cancer cell lines, MCF7 and T-47D, but higher than that in MDA-MB-231 (Fig. 3b). In luminal cell lines, CK18 was highly expressed but was absent in CGBC 01 and CGBC 02 (Fig. 3b). CK19 in our cell lines was as weakly expressed as in MCF 10A. Some markers expressed in the primary tumors changed after in vitro culture or isolation procedures. For example, the strong expression of PR, CK18 and CK19 in tumor tissue declined in both the established cell lines (Figs. 1, 3b). In contrast, the expression of basal-cell markers (CK5, CK6 and EGFR) became stronger in both CGBC 01 and CGBC 02 cells, which was not observed in luminal type and HER2-enriched (MDA-MB 453) cells (Fig. 3b). E-cadherin was also expressed in CGBC 01 and CGBC 02, as was consistent with immunohistochemistry staining results of their primary tissue (Fig. 3b). In conclusion, CGBC 01 and CGBC 02 expressed protein markers of basal-like breast cancer cells.

### Cell line authentication and genotyping

**Fig. 4:**
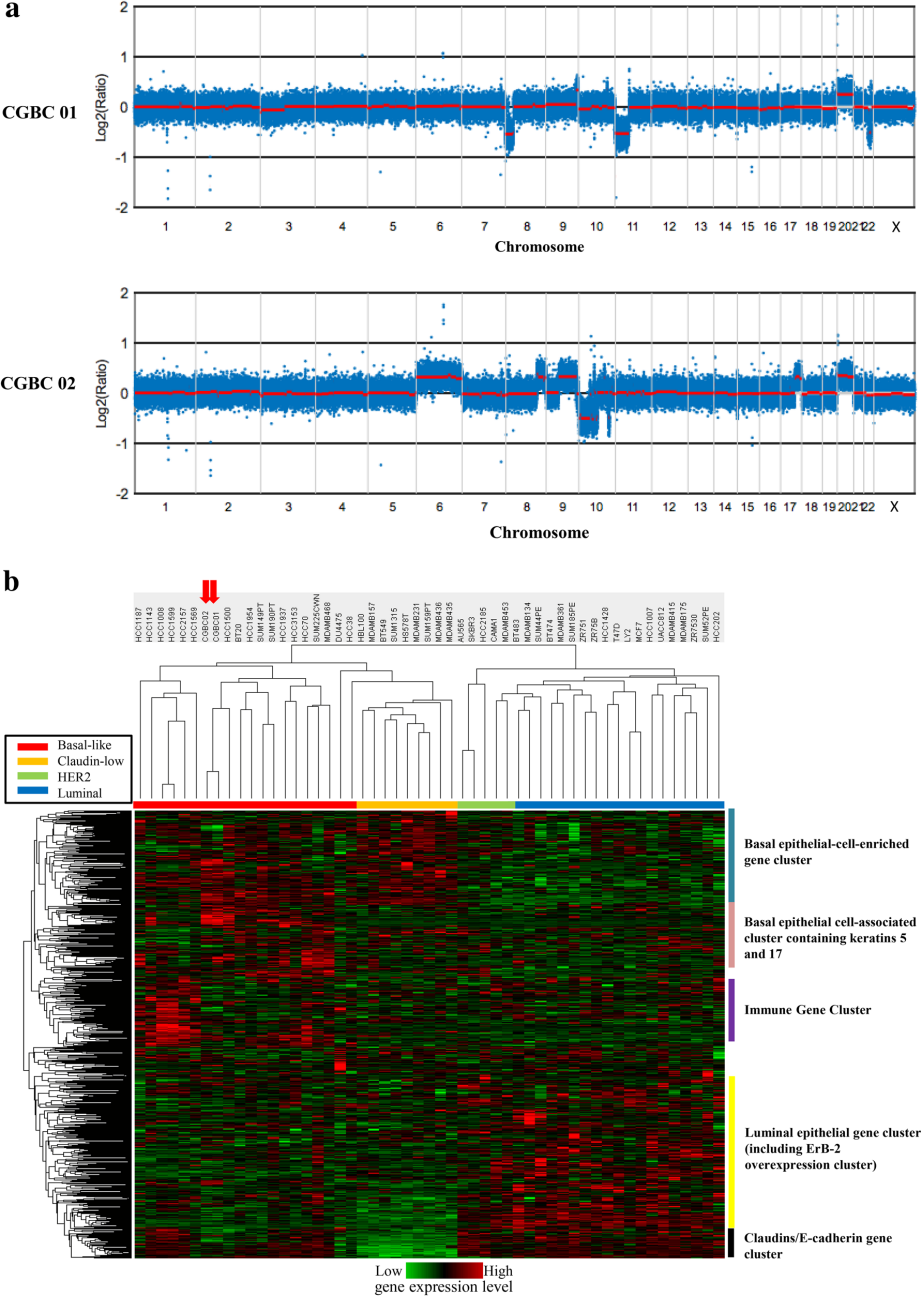
Genome-wide copy number alteration and gene expression profile analysis of CGBC 01 and CGBC 02 cells. **a** The genomic landscape of CGBC 01 and 02 cells assessed by Affymetrix SNP 6.0 array. Chromosomal copy number changes and loss of heterozygosity were analyzed using Chromosome Analysis Suite (ChAS; Affymetrix; Thermo Fisher Scientific Inc.). Relative genomic position is shown on the *x*-axis and the log2 ratio of normalized intensity with respect to normal references is shown on the left *y*-axis. Red line: smoothing moving average of calibrate copy number estimate. **b** Classification of constitutive gene expression profiles of CGBC 01 and CGBC 02 based on the expression database of breast cancer cells by hierarchical clustering [3, 38]. The highest expression level (in log2 space) for each cell fraction is shown in red, average expression in black, and lowest expression in green

Short tandem repeat (STR) profiles of CGBC 01 and CGBC 02 (Supplementary Table 3) were not identical to each other, but were identical to the profiles of their original tumor tissue. The cell lines were further authenticated by the absence of a match of their profile with any other cell lines such as Hela cells in ATCC public database. Genome-wide Affymetrix SNP 6.0 array was used for additional genotyping of CGBC 01 and CGBC 02. The ethnic origin of these two cell lines was revealed based on the reported race-distinguishing single nucleotide polymorphisms (SNPs); including rs11051 (G/A, -strand) to distinguish between “CHB and YRI”, rs489095 (A/G) for “CHB and CEU”, rs6546753 (G/T) for “JPT and YRI”, rs6437783 (C/T) for “JPT and CEU”, and rs735480 (C/T) for “YRI and CEU [36, 37]. The SNP status of both cell lines was compatible with the signatures of Han-Chinese in Beijing (CHB) (Supplementary Table 4). The copy number changes identified by Affymetrix SNP 6.0 array included gains at chromosome 9q and 20, and losses at 8p, 11p, and 22q in CGBC 01; gains at chromosomes 6, 8q, 9q, 17q and 20, and losses at chromosome 10 in CGBC 02 (Fig. 4a). The allelic loss, gain and heterozygosity in CGBC 01 and CGBC 02 cells are summarized in Supplementary Table 5.

### Molecular subtype

Gene expression profiles of CGBC 01 and CGBC 02 were assessed with Affymetrix Human Genome U133 Plus 2.0 Array. Gene expression profiles of commonly used human breast cancer cell lines were downloaded from GEO (Gene Expression Omnibus) dataset accession GSE15376 [3]. Gene expression signatures of CGBC 01 and CGBC 02 were clustered with those of basal-like breast cancer cell lines [3, 38] (Fig. 4b).

### Colony formation

**Fig. 5:**
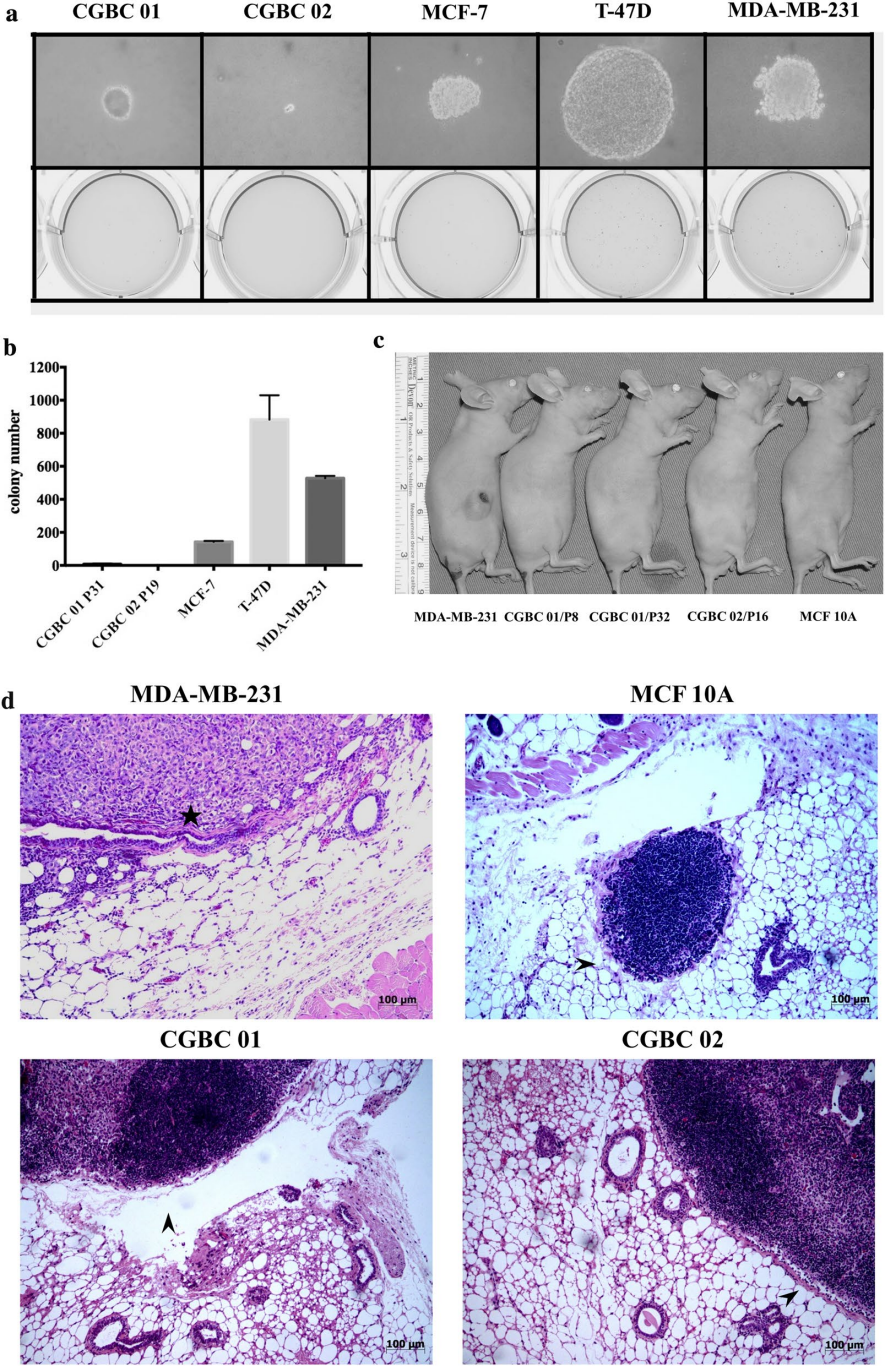
In vitro and in vivo tumorigenicity of CGBC 01 and CGBC 02. Clonogenic assay of cell lines in soft agar after 21–28 days in culture. **a** The representative colonies are shown at ×400 light fields. **b** Average number of colonies per plate in three replicates for each cell line. **c** Tumor formation of cell lines in the 4th mammary fat pads of nude mice 4 months after injection. **d** Microscopic view of injected mouse mammary gland confirmed no cancer growth after injection with CGBC 01, CGBC 02 and MCF10A. Tumor growth of MDA-MB-231 was present (black star). The cell-rich areas are intramammary lymph nodes (black arrow)

Colony formation was evaluated in the soft agar seeded with CGBC 01 and CGBC 02 cells after being cultured for 21–28 days. There were a few tiny colonies observed in CGBC 01 culture, whereas CGBC 02 barely grew into any colony. The colony size of CGBC 01 was significantly smaller than those of MCF7, T-47D, and MDA-MB-231 (Fig. 5a). The number of colonies formed by CGBC 01 and CGBC 02 was significantly smaller than that of the three reference cell lines (Fig. 5b).

### Mice xenograft

To observe in vivo tumorigenicity, 5 × 10^6^ cells from each cell line were injected into 4th mammary fat pad of female BALB/c nude mice at the age between 5 and 8 weeks. Approximately 4 weeks after injection, bumps due to local reaction subsided and growth of tumor nodules became apparent. Tumor formation was prominent only in mice injected with MDA-MB-231, but not in any of the mice injected with other cell lines including CGBC 01, CGBC 02, and MCF 10A (Fig. 5c). Growth of cancer xenografts were observed for 4 months, till the diameter of the largest subcutaneous tumor formed from MDA-MB-231 reached 1.5 cm. Liver and lung were carefully explored after killing. No distant metastatic tumor foci were found either by gross or microscopic examination in the mice injected with CGBC 01 and CGBC 02. Our data suggest that the two cell lines, CGBC 01 and CGBC 02 have very low tumorigenicity (Fig. 5c, d). Table 2 summarizes the phenotypes and genotypes of CGBC 01 and CGBC 02.

**Table 2:** Summary of expressed markers and molecular subtypes of CGBC01, CBGC02 and standard breast cancer cell lines

Markers	CGBC01	CGBC02	MDA-MB-231	T-47D	MCF7	MDA-MB-453	MCF 10A	Detecting method
EpCAM	Strongly positive	Strongly positive	Weak	Strongly positive	NA	NA	NA	FCM
ER	Weak	Negative	Negative	Strongly positive	Positive	Negative	Negative	WB
PR	Weak	Weak	Extremely weak	Strongly positive	Positive	Extremely weak	Weak	WB
HER2	Extremely weak	Negative	Extremely weak	Extremely weak	Negative	Strongly positive	Negative	WB
CK18	Negative	Negative	Extremely weak	Strongly positive	Strongly positive	Positive	Negative	WB
CK19	Extremely weak	Extremely weak	Strongly positive	Strongly positive	Strongly positive	Strongly positive	Negative	WB
CK5	Positive	Strongly positive	Negative	Negative	Negative	Negative	Extremely weak	WB
CK6	Positive	Strongly positive	Negative	Negative	Negative	Negative	Negative	WB
E-cadherin	Positive	Positive	Negative	Positive	Positive	Positive	Positive	WB
EGFR	Positive	Extremely weak	Strongly positive	Extremely weak	Negative	Negative	Extremely weak	WB
Molecular subtype	Basal-like	Basal-like	Claudin-low	Luminal A	Luminal A	HER2	NA	Microarray

*FCM* flow cytometry, *WB* western blot, *ER* estrogen receptor, *PR* progesterone receptor, *HER2* erbB-2 receptor tyrosine kinase 2,* CK18* cytokeratin 18,* CK19* cytokeratin 19,* CK5* cytokeratin 5,* CK6* cytokeratin 6,* EGFR* epidermal growth factor receptor,* NA* not applicable for detection

## Discussion

In this study, we totally enrolled 33 breast cancer patients and collected 50 cancer/normal tissue specimens for established cell lines (Supplementary Table 2). Among the 33 cancer samples enrolled, only CGBC 01 (from patient M_17) and CGBC 02 (from patient M_18) were able to propagate spontaneously after the 10^th^ passage, other primary cultures failed to maintain continuous proliferation either due to fibroblast outgrowth in early passages or senescence after the 10^th^ passage in vitro. The success rate of established cell lines using reported method [31, 32] was less than 10% in our study. The two samples developed into CGBC 01 and CGBC 02 were both strongly positive for PR, not HER2-amplified, and very weak or absent in ER expression, which is distinguishable from all other failed samples. Furthermore, the epithelial cell population was enriched by selection with EpCAM-conjugated beads [31, 33] and the use of serum-free culture medium [39]. Although beads separation allows quick enrichment of target population, the shear force encountered by cells during the process might cause injury to the eluted cells and predispose to later senescence that might partially explain the low success rate but development of highly enriched EpCAM-positive cell lines using our method.

The expression of PR, CK18, and CK19 was significantly different between primary tumors and the established cell lines, especially after EpCAM selection (Figs. 1, 3). The loss of PR, CK18, and CK19 can be related to tissue dissociation procedure, in vitro cell culture, EpCAM enrichment process, or selection pressure from in vitro culture environment. Recent reports showed that EpCAM selection results in the loss of claudin-low cell population [12], indicating that EpCAM is not a universal marker of all breast cancer cells. EpCAM selection could enrich certain populations of breast cancer subtypes that possess low tumorigenicity. On the other hand, intratumoral heterogeneity also contributes to the change in the expression profile in cell culture. In vitro culture may provide different growth advantages for cells of small population in the original heterogeneous tumor. Recovering primary cell cultures from earlier passages may help developing cell lines from same origin but with other phenotypes. Among all the markers we analyzed, the expression of EGFR in CGBC 01 was the most interesting, as it was not present on the original tumor by IHC. In addition to the influence from in vitro processing, one other possible explanation was the expression of EGFR being induced by the supplement with recombinant hEGF in the culture media. The enhancement of basal-cell markers (CK5 and CK6) in CGBC 01 and CGBC 02 could be due to all the reasons mentioned above. Therefore, it is worth investigating if the phenotypes change with different culture supplements or FBS.

Genome-wide copy number alteration map showed that chromosome 20 was amplified in both cell lines (Fig. 4a). Amplification of chromosome 20q may occur in early tumorigenic transformation and initiate tumor formation [40]. This chromosome also harbors several loci reported to be cell line-specific recurrent genomic gains such as 20q11 and 20q13 [41] and driver genes of breast cancer pathogenesis [34]. Indeed, gains is 20q13 were present in both CGBC 01 and CGBC 02 (Supplementary Table 5). Gain at 8q24.3 and loss at 8p23.3–p21.2 is associated with resistance to taxane-based neoadjuvant chemotherapy in ductal breast cancer [42]. Based on gene expression signatures [3], our results suggested that CGBC 01 and CGBC 02 are basal-like breast cancer cell lines (Fig. 4b). The high expression of CK5/6 and EGFR but low ER/PR and CK18 is compatible with basal-like breast cancer features [38, 43, 44]. The non-tumorigenic characteristics of our cell lines represent cancer cells of less aggressiveness, which allows researchers the advantage of observing the functional change related to specific genetic manipulation in the earlier stages of tumorigenesis.

Asian breast cancer is different from Western breast cancer by earlier age at onset, affecting more premenopausal women. The postmenopausal incidence maintains a plateau instead of steadily increasing after the age of 50, which was common in Caucasian women. The distinctive epidemiological trend was widely observed across Eastern Asia with high to progressively lower incidence along the south-to-north gradient [20, 22, 24]. The incidence of female invasive breast cancer in Taiwan is 70.74 cases per 10,000 women by year 2014 according to Taiwan Cancer Registry Database (http://tcr.cph.ntu.edu.tw/main.php?Page=A5B2). The incidence is expected to rise in the next 10 years, and the effect of birth cohort reflecting Westernized lifestyle bears the blame [22, 45, 46]. However, in stage II/III breast cancers, Asian and Pacific Island women have better breast cancer-specific survival than white and African-American women even after adjusting for factors including screening, tumor biology and treatment [47]. In Taiwan, there is a higher prevalence (67%) of favorable molecular subtype, luminal A, in premenopausal breast cancer, but lower prevalence of the more aggressive basal-like subtype (9% before age 50 and 17% after age 50) as in premenopausal African-American women (39%) [28]. We are not clear whether genetic factors contribute to the difference in carcinogenesis. Non-luminal breast cancer cell lines like CGBC 01 and CGBC 02 may help clarify the ethnic difference in Asian breast cancer carcinogenesis.

## Conclusion

Two cell lines, CGBC 01 and CGBC 02, were established from premenopausal breast cancer patients in Taiwan with Han-Chinese genetic background. The molecular signatures of both cells were categorized into basal-like subtype and expressed basal markers. Their proliferation rate was moderate in vitro and showed low tumorigenicity when transplanted into the mammary gland of nude mice.

## Electronic supplementary material

Below is the link to the electronic supplementary material.

Supplementary material 1 (DOCX 79 kb)
