# Supplementary material for: Establishment of two basal-like breast cancer cell lines with extremely low tumorigenicity from Taiwanese premenopausal women
Source: Hum Cell. 2018 Feb 26;31(2):154–66. doi: 10.1007/s13577-017-0197-3 (PMC5852199; doi:10.1007/s13577-017-0197-3)
Supplement: Supplementary file 1 — Supplementary material 1 (DOCX 79 kb) [file 13577_2017_197_MOESM1_ESM.docx]

**SUPPLEMENTARY DATA**

**Supplementary Table 1. List of human breast cancer cell lines available in ATCC**

| Name | Reference Source | Molecular Subtype | Tissue Source | Ethnics | Age |
| --- | --- | --- | --- | --- | --- |
| HCC1187 | ATCC® CRL-2322™ | basal-like | mammary gland; breast | Caucasian, White | 41 |
| HCC1143 | ATCC® CRL-2321™ | basal-like | breast; mammary gland/duct | Caucasian | 52 |
| HCC1008 | ATCC® CRL-2320™ | basal-like | breast; mammary gland/duct; derived from metastatic site: lymph node | Black | NA |
| HCC1599 | ATCC® CRL-2331™ | basal-like | mammary gland; breast/duct | Caucasian, White | NA |
| HCC2157 | ATCC® CRL-2340™ | basal-like | mammary gland; breast | Black | 48 |
| HCC1569 | ATCC® CRL-2330™ | basal-like | mammary gland; breast | Black | 70 |
| CGBC02 |  | basal-like | mammary gland; breast | Chinese, Han | 44 |
| CGBC01 | BCRC No.60610 | basal-like | mammary gland; breast | Chinese, Han | 46 |
| HCC1500 | ATCC® CRL-2329™ | basal-like | breast; mammary gland/duct | Black | 32 |
| BT-20 | ATCC® HTB-19™ | basal-like | mammary gland/breast | Caucasian | 74 |
| HCC1954 | ATCC® CRL-2338™ | basal-like | mammary gland; breast/duct | East Indian | 61 |
| SUM149PT | Asterand SUM-149PT | basal-like | inflammatory ductal carcinoma; breast | NA | NA |
| SUM190PT | Asterand SUM-190PT | basal-like | inflammatory carcinoma; breast | NA | NA |
| HCC1937 | ATCC® CRL-2336™ | basal-like | mammary gland; breast/duct | Caucasian | 24 |
| HCC3153 | Adi Gazdar, University of Texas-Southwestern Medical Center | basal-like | breast; mammary gland/duct | NA | NA |
| HCC70 | ATCC® CRL-2315™ | basal-like | breast; mammary gland/duct | Black | 49 |
| SUM225CWN | Asterand SUM-225CWN | basal-like | chest wall nodule, metastatic, mammary ductal cacrinoma | NA | NA |
| MDA-MB-468 | ATCC® HTB-132™ | basal-like | derived from metastatic site: pleural effusion | Black | 51 |
| DU4475 | ATCC® HTB-123™ | basal-like | mammary gland; breast; derived from metastatic site: skin carcinoma | Caucasian | NA |
| HCC38 | ATCC® CRL-2314™ | basal-like | mammary gland; breast/duct | Caucasian, White | 50 |
| HBL-100 | CLS Cell Lines Service 300178 | caudin-low | normal mammary gland; breast | Caucasian | 27 |
| MDA-MB-157 | ATCC® HTB-24™ | caudin-low | mammary gland; breast/medulla | Black | 44 |
| BT-549 | ATCC® HTB-122™ | caudin-low | mammary gland; breast | Caucasian | 72 |
| SUM1315MO2 | University of Michigan (http://www.cancer.med.umich.edu/breast_cell/Production/index.html) | caudin-low | derived from metastatic site: skin | NA | NA |
| Hs 578T | ATCC® HTB-126™ | caudin-low | mammary gland/breast | Caucasian | 74 |
| MDA-MB-231 | ATCC® HTB-26™ | caudin-low | derived from metastatic site: pleural effusion | Caucasian | 51 |
| SUM159PT | Asterand SUM-159PT | caudin-low | mammary gland, anaplastic carcinoma | NA | NA |
| MDA-MB-436 | ATCC® HTB-130™ | caudin-low | derived from metastatic site: pleural effusion | Caucasian | 43 |
| MDA-MB-435 | ATCC® HTB-129™ | caudin-low | derived from metastatic site: pleural effusion | Caucasian, White | 31 |
| AU565 | ATCC® CRL-2351™ | HER2 | derived from metastitic site: malignant pleural effusion | Caucasian, White | 43 |
| SK-BR-3 | ATCC® HTB-30™ | HER2 | derived from metastatic site: pleural effusion | Caucasian | 43 |
| HCC2185 | HMS LINCS ID:51093 | HER2 | breast ductal carcinoma/breast | white | 49 |
| CAMA-1 | ATCC® HTB-21™ | HER2 | derived from metastatic site: pleural effusion | Caucasian | 51 |
| MDA-MB-453 | ATCC® HTB-131™ | HER2 | derived from metastatic site: pericardial effusion | Caucasian | 48 |
| BT-483 | ATCC® HTB-121™ | luminal | mammary gland; breast | Caucasian | 23 |
| MDA-MB-134-VI | ATCC® HTB-23™ | luminal | derived from metastatic site: pleural effusion | Caucasian | 47 |
| SUM44PE | Asterand SUM-44PE | luminal | breast carcinoma/breast | NA | NA |
| BT-474 | ATCC® HTB-20™ | luminal | mammary gland; breast/duct | Caucasian | 60 |
| MDA-MB-361 | ATCC® HTB-27™ | luminal | derived from metastatic site:brain | Caucasian | 40 |
| SUM185PE | Asterand SUM-185PE | luminal | derived from metastatic site: pleural effusion | NA | NA |
| ZR-75-1 | ATCC® CRL-1500™ | luminal | derived from metastatic site: ascites | Caucasian | 63 |
| ZR75B | Mark Lippman (National Cancer Institute) | luminal | derived from metastatic site: ascites | Caucasian | NA |
| HCC1428 | ATCC® CRL-2327™ | luminal | derived from metastatic site: adenocarcinoma and pleural effusion | Caucasian, White | 49 |
| T-47D | ATCC® HTB-133™ | luminal | derived from metastatic site: pleural effusion | NA | 54 |
| LY2 | Mark Lippman (National Cancer Institute) | luminal | derived from metastatic site: pleural effusion | Caucasian, White | 69 |
| MCF7 | ATCC® HTB-22™ | luminal | derived from metastatic site: pleural effusion | Caucasian | 69 |
| HCC-1007 | AcceGen :ABC-TC0294 | luminal | mamamry ductal caarcnoma; breast | Black | 67 |
| UACC-812 | ATCC® CRL-1897™ | luminal | mamamry ductal caarcnoma; breast | NA | 42 |
| MDA-MB-415 | ATCC® HTB-128™ | luminal | derived from metastatic site: pleural effusion | Caucasian, White | 38 |
| MDA-MB-175-VII | ATCC® HTB-25™ | luminal | derived from metastatic site: pleural effusion | Black | 56 |
| ZR-75-30 | ATCC® CRL-1504™ | luminal | derived from metastatic site: ascites | Black | 47 |
| SUM52PE | Asterand SUM-52PE | luminal | derived from metastatic site: pleural effusion | NA | NA |
| HCC202 | ATCC® CRL-2316™ | luminal | breast; mammary gland/duct | Caucasian, White | NA |

**Supplementary Table 2. Demography data of enrolled patients for establishment primary cell cultures in this study**

| **Patient Number** | **Age** | **Diagnosis** | **Tissue used** | **Stage** | **Grade^#^** | **ER^#^**  **(% positive)** | **PR^#^**  **(% positive)** | **HER2 amplification^#*^** |
| --- | --- | --- | --- | --- | --- | --- | --- | --- |
| M_1 | 45 | IDC | Breast tumor | IIIA | 1 | 30 | 40 | + |
| M_2 | 58 | IDC | Breast tumor | IA | 1 | 0 | 0 | + |
| M_3 | 48 | IDC | Breast tumor, and non-tumor | IIB | 1 | 35 | 65 | + |
| M_4 | 33 | IDC | Breast tumor, and non-tumor | IIIC | 2 | 50 | 10 | + |
| M_5 | 28 | IDC | Breast tumor, and non-tumor | IA | NA | 90 | 90 | - |
| M_6 | 53 | IDC | Breast tumor | IIIA | 2 | 60 | 60 | - |
| M_7 | 49 | IDC | Breast tumor | IIIA | 2 | 20 | 10 | + |
| M_8 | 52 | IDC | Breast tumor^+^ | IV | NA | 80 | 80 | + |
| M_9 | 26 | IDC | Breast tumor | IIA | 3 | 80 | 20 | + |
| M_10 | 39 | IDC | Breast tumor | IIA | 3 | 0 | 0 | - |
| M_11 | 48 | IDC | Breast tumor^+^ | IIIA | 2 | 70 | 6 | - |
| M_12 | 63 | IDC | Breast tumor, and non-tumor | IIB | 1 | 90 | 90 | + |
| M_13 | 61 | IDC | Breast tumor | IIIC | 2 | 0 | 0 | NA |
| M_14 | 36 | IDC | Breast tumor^+^ | IIA | 3 | 0 | 0 | - |
| M_15 | 60 | IDC | Breast tumor | IA | 3 | 0 | 0 | + |
| M_16 | 48 | IDC | Breast tumor | IIA | 2 | 80 | 85 | - |
| M_17 | 46 | IDC | Breast tumor, and non-tumor | IIIC | 2 | 0 | 80 | - |
| M_18 | 44 | IDC | Breast tumor, and non-tumor | IIA | 3 | 20 | 99 | - |
| M_19 | 51 | IDC | Breast tumor | IIA | 3 | 0 | 0 | + |
| M_20 | 53 | IDC | Breast tumor, and non-tumor | IIA | 2 | 0 | 0 | + |
| M_21 | 38 | IDC | Breast tumor, and non-tumor | IIIA | 3 | 0 | 0 | + |
| M_22 | 28 | IDC | Breast tumor, and non-tumor | IIIC | 3 | 60 | 95 | - |
| M_23 | 57 | IDC | Breast tumor | IIIC | 3 | 30 | 1 | + |
| M_24 | 34 | IDC | Breast tumor, and non-tumor | IIB | 3 | 75 | 65 | + |
| M_25 | 78 | IDC | Breast tumor | IIB | 2 | 90 | 90 | - |
| M_26 | 71 | IDC | Breast tumor | IIIC | 3 | 50 | 60 | - |
| M_27 | 65 | IDC | Breast tumor | 0 | low | 90 | 50 | - |
| M_28 | 49 | IDC | Breast tumor, and non-tumor | IIA | 3 | 0 | 0 | - |
| M_29 | 48 | IDC | Breast tumor, and non-tumor | IIIA | 1 | 60 | 60 | - |
| M_30 | 55 | IDC | Breast tumor, and non-tumor | IIA | 2 | 85 | 90 | - |
| PE_1 | 46 | IDC | Metastatic pleural effusion | IV | 2 | 0 | 0 | + |
| PE_2 | 55 | IDC | Metastatic pleural effusion | IV | 3 | 0 | 0 | + |
| PE_3 | 49 | IDC | Metastatic pleural effusion | IV | 2 | 80 | <5 | + |
| B_1 | 18 | Fibroadenoma | Normal breast tissue next to fibroadenoma | NA | NA | NA | NA | NA |
| B_2 | 35 | Fibroadenoma | Normal breast tissue next to fibroadenoma | NA | NA | NA | NA | NA |
| B_3 | 32 | Fibroadenoma | Normal breast tissue next to fibroadenoma | NA | NA | NA | NA | NA |
| B_4 | 66 | No pathologic diagnosis | Normal breast tissue at reduction mammoplasty | NA | NA | NA | NA | NA |

Annotations: **^#^**Grade, ER, PR, and HER2 level refers to the status of the primary breast tumor at diagnosis; ^+^Tumor samples were collected after neoadjuvant chemotherapy; ^*^ HER2 amplification: more than 30% strong membrane staining by IHC or ratio more than 2.2 by fluorescence *in situ* hybridization

Abbreviations: IDC: invasive ductal carcinoma; DCIS: ductal carcinoma in situ; ER: estrogen receptor; PR: progesterone receptor; HER2: erbB2; NA: not available

**Supplementary Table 3. STR profile of CGBC 01 and CGBC 02**

| Locus | CGBC 01 (P17) | CGBC 02 (P16) |
| --- | --- | --- |
| D8S1179 | 13,14 | 10, 14 |
| D21S11 | 30, 32.2 | 29, 31 |
| D7S820 | 9, 11 | 12 |
| CSF1PO | 10, 13 | 12 |
| D3S1258 | 15, 16 | 16, 17 |
| TH01 | 6, 8 | 9 |
| D13S317 | 8, 9 | 10, 12 |
| D16S539 | 11, 13 | 12, 13 |
| D2S1338 | 23, 24 | 17, 22 |
| D19S433 | 14 | 13.2, 15.2 |
| vWA | 17, 18 | 17, 18 |
| TPOX | 8 | 9, 11 |
| D18S51 | 13, 14 | 13, 15 |
| Amelogenin | X | X |
| D5S818 | 9, 12 | 9, 13 |
| FGA | 23, 24 | 23, 24 |

**Supplementary Table 4. The ethnicity-specific SNP patterns of CGBC 01 and CGBC 02**

| Probe Set ID | dbSNP RS ID | Chromosome | Physical Position | Strand | Cytoband | Allele A | Allele B | CGBC 01 | CGBC 02 | Ethnics |
| --- | --- | --- | --- | --- | --- | --- | --- | --- | --- | --- |
| SNP_A-2035146 | rs11051^*^ | 4 | 42089177 | - | p13 | C | T | BB | BB | CHB |
| SNP_A-2281582 | rs489095^*^ | 5 | 42089177 | + | p15.32 | A | G | AA | AA | CHB |
| SNP_A-8516916 | rs6437783^*^ | 3 | 108172817 | + | q13.13 | C | T | AA | AA | CHB |
| SNP_A-1826754 | rs6546753^*^ | 2 | 72490319 | + | p13.2 | G | T | AA | AA | Asian^#^ |

*rs11051 (G/A) for “CHB and YRI”, rs489095 (T/C) for “CHB and CEU”, rs6546753 (G/T) for “JPT and YRI”, rs6437783 (C/T) for “JPT and CEU”, and rs735480 (C/T) for “YRI and CEU (Abbreviations: CHB: Han Chinese persons in Beijing; JPT: Japanese persons in Tokyo; YRI: African marriage pairs from Yoruba in Ibadan; CEU: Caucasian marriage pairs of European descent resided in Utah) [36, 37]

# Asian: CHB and JPT

**Supplementary Table 5. Regions of copy number change and loss of heterozygosity in CGBC 01 and CGBC 02 assessed by SNP 6.0 array**

| sAMPLES | PHYSICAL POISTION | Cytoband | Type |
| --- | --- | --- | --- |
| CGBC 01 | chr20:1665232-62459663 | p13-q13.33 | Gain |
| CGBC 01 | chr9:131086548-141004479 | q34.11-q34.3 | Gain |
| CGBC 01 | chr11:336191-49686198 | p15.5-p11.12 | Loss |
| CGBC 01 | chr22:32975658-41850500 | q12.3-q13.2 | Loss |
| CGBC 01 | chr8:46385-27446900 | p23.3-p21.1 | Loss |
| CGBC 01 | chr11:198509-46187583 | p15.5-p11.2 | LOH |
| CGBC 01 | chr22:36460073-42356886 | q12.3-q13.2 | LOH |
| CGBC 01 | chr8:161221-27758391 | p23.3-p21.1 | LOH |
| CGBC 01 | chr8:49220070-52133622 | q11.21 | LOH |
| CGBC 01 | chrX:55361709-85473228 | p11.21-q21.2 | LOH |
| CGBC 02 | chr17:55701133-81049726 | q22q23.2-q25.3 | Gain |
| CGBC 02 | chr20:61292-62916056 | p13-q13.33 | Gain |
| CGBC 02 | chr6:149648-170982522 | p25.3-q27 | Gain |
| CGBC 02 | chr8:112539455-146298143 | q23.3-q24.3 | Gain |
| CGBC 02 | chr9:68725185-131425764 | q21.11-q34.11 | Gain |
| CGBC 02 | chr10:109599388-113963806 | q25.1-q25.2 | Loss |
| CGBC 02 | chr10:150531-38569877 | p15.3-p11.1 | Loss |
| CGBC 02 | chr10:47964179-58167465 | q11.22-q21.1 | Loss |
| CGBC 02 | chr10:64256829-70061439 | q21.2-q21.3 | Loss |
| CGBC 02 | chr10:2787285-14088714 | p15.3-p13 | LOH |
| CGBC 02 | chr10:33430296-36325494 | p11.22-q21.1 | LOH |
| CGBC 02 | chr8:49220070-52147041 | q11.21 | LOH |
| CGBC 02 | chrX:55351390-85473228 | p11.21-q21.2 | LOH |

**Supplementary Figure 1. Proliferation curve of CGBC 01 and CGBC 02 with standard cell lines**

Proliferation curves of CGBC 01(P53), CGBC 02(P50) and standard cell lines. All cell lines were cultured in appropriate culture media and incubator for 120 hrs. The number of living cells were determined using Cell Counting Kit-8 (CCK-8) by the measurement of tetrazolium salt, WST-8, is reduced by dehydrogenase activities in cells to give a yellow-color formazan dye dissolved in culture media.
